# Transparent Reporting of AI in Systematic Literature Reviews: Development of the PRISMA-trAIce Checklist

**DOI:** 10.2196/80247

**Published:** 2025-12-10

**Authors:** Dirk Holst, Keno Moenck, Julian Koch, Ole Schmedemann, Thorsten Schüppstuhl

**Affiliations:** 1 Institute of Aircraft Production Technology, Hamburg University of Technology, Am Schwarzenberg-Campus 1, Hamburg, 21073, Germany, 49 17689103318, 49 40427314551

**Keywords:** systematic literature review, reporting guideline, artificial intelligence, AI, large language models, Preferred Reporting Items for Systematic Reviews and Meta-Analyses, PRISMA, transparency, evidence synthesis

## Abstract

**Background:**

Systematic literature reviews (SLRs) build the foundation for evidence synthesis, but they are exceptionally demanding in terms of time and resources. While recent advances in artificial intelligence (AI), particularly large language models, offer the potential to accelerate this process, their use introduces challenges to transparency and reproducibility. Reporting guidelines such as the PRISMA-AI (Preferred Reporting Items for Systematic Reviews and Meta-Analyses–Artificial Intelligence Extension) primarily focus on AI as a subject of research, not as a tool in the review process itself.

**Objective:**

To address the gap in reporting standards, this study aimed to develop and propose a discipline-agnostic checklist extension to the PRISMA 2020 statement. The goal was to ensure transparent reporting when AI is used as a methodological tool in evidence synthesis, fostering trust in the next generation of SLRs.

**Methods:**

The proposed checklist, named PRISMA-trAIce (PRISMA–Transparent Reporting of Artificial Intelligence in Comprehensive Evidence Synthesis), was developed through a systematic process. We conducted a literature search to identify established, consensus-based AI reporting guidelines (eg, CONSORT-AI [Consolidated Standards of Reporting Trials–Artificial Intelligence] and TRIPOD-AI [Transparent Reporting of a Multivariable Prediction Model of Individual Prognosis or Diagnosis–Artificial Intelligence]). Relevant items from these frameworks were extracted, analyzed, and thematically synthesized to form a modular checklist that integrated with the PRISMA 2020 structure.

**Results:**

The primary result of this work is the PRISMA-trAIce checklist, a comprehensive set of reporting items designed to document the use of AI in SLRs. The checklist covers the entire structure of an SLR, from title and abstract to methods and discussion, and includes specific items for identifying AI tools, describing human-AI interaction, reporting performance evaluation, and discussing limitations.

**Conclusions:**

PRISMA-trAIce establishes an important framework to improve the transparency and methodological integrity of AI-assisted systematic reviews, enhancing the trust required for the responsible application of AI-assisted systematic reviews in evidence synthesis. We present this work as a foundational proposal, explicitly inviting the scientific community to join an open science process of consensus building. Through this collaborative refinement, we aim to evolve PRISMA-trAIce into a formally endorsed guideline, thereby ensuring the collective validation and scientific rigor of future AI-driven research.

## Introduction

Evidence-based practice is the cornerstone of modern science and decision-making across a wide range of disciplines, from clinical medicine and social sciences to engineering and computer science. One method of generating this evidence is a systematic literature review (SLR) [1,2]. In contrast to narrative reviews, SLRs follow a rigorous and predefined methodology to identify, appraise, and synthesize the totality of relevant research on a specific question. The goal is to minimize author bias and provide a reliable answer to the research question through a comprehensive and reproducible process [2].

Despite its widely recognized value, conducting an SLR presents considerable challenges. The requirement for completeness necessitates extensive searches across various scientific databases, often yielding thousands of potentially relevant publications. Each of these must then be manually screened for relevance, typically by at least 2 independent reviewers. This is followed by detailed data extraction and quality assessment of the included studies. The process is not only methodologically demanding but also extremely time-consuming and labor intensive, which significantly hinders the broad application of SLR methodology outside well-funded research groups [3].

Recent breakthroughs in natural language processing, particularly the development and wide availability of large language models such as GPT-4 or Claude 3, offer new possibilities to overcome these challenges. Today, artificial intelligence (AI)–supported tools can assist in almost all phases of the SLR process [4,5], from generating and optimizing search strategies to semiautomating the screening of titles and abstracts and extracting structured data from full texts [5,6]. Studies indicate that these technologies can reduce the manual workload by 50% to 75% [7], bringing the promise of democratizing SLR methodology within reach [8].

However, the integration of AI tools into the scientific process poses a new, fundamental challenge: ensuring transparency, traceability, and trust in the results [9]. The “black box” nature of many LLMs and their known weaknesses, such as “hallucinations” [10,11] or the reproduction of bias from training data [12,13], stand in direct contradiction to the core principles of SLRs. To ensure trust in traditional SLRs, the scientific community relies on established reporting guidelines, most notably the PRISMA (Preferred Reporting Items for Systematic Reviews and Meta-Analyses) 2020 statement, which mandates detailed documentation of each step in the SLR process [14].

An analogous guideline including the use of AI in SLRs is clearly needed. Although a corresponding extension, the PRISMA-AI (PRISMA–Artificial Intelligence Extension), was announced in 2022, it has not yet been published. More importantly, according to the planning document, its conceptual focus is primarily on reporting SLRs that investigate AI as a subject of research in clinical medicine [15]. There is still a complete lack of a guideline for using AI as a tool within the research process applicable across all disciplines and an urgent need to formulate globally recognized guidelines for the use of AI tools [16]. This gap creates the risk of AI-supported SLRs becoming a nonstandardized and nontransparent method and therefore scientifically questionable.

To close this reporting gap and, thereby, mitigate the associated risks, we propose PRISMA-trAIce (PRISMA–Transparent Reporting of Artificial Intelligence in Comprehensive Evidence Synthesis). This checklist synthesizes key principles from established AI reporting guidelines to provide a structured and well-founded starting point. We offer this framework as a catalyst for community discussion, aiming to accelerate the collaborative development of a formal, consensus-based standard for transparent AI reporting in SLRs.

## Methods

### Formulation of the PRISMA-trAIce Checklist

We developed the PRISMA-trAIce checklist through a systematic, multistage process.

First, our targeted literature review focused on established, consensus-based AI reporting guidelines, primarily sourced from the EQUATOR (Enhancing the Quality and Transparency of Health Research) Network, a recognized authority on such standards [17-22]. Our search included keywords such as “artificial intelligence,” “machine learning,” and “reporting guideline.” The methodological approach was designed to build on robust, existing scientific consensus established by leading reporting guidelines. Many of these foundational guidelines, such as the CONSORT-AI (Consolidated Standards of Reporting Trials–Artificial Intelligence) and TRIPOD-AI (Transparent Reporting of a Multivariable Prediction Model of Individual Prognosis or Diagnosis–Artificial Intelligence), were chosen as they represent the most widely adopted, cross-domain standards for research involving AI, thus providing the most robust and consensus-driven foundation for our synthesis. From this search, the following core guidelines were selected for adaptation: CONSORT-AI [18], SPIRIT-AI (Standard Protocol Items: Recommendations for Interventional Trials–Artificial Intelligence) [19], TRIPOD-AI and TRIPOD-LLM (Transparent Reporting of a Multivariable Prediction Model of Individual Prognosis or Diagnosis–Large Language Model) [20,23], DECIDE-AI (Developmental and Exploratory Clinical Investigations of Decision Support Systems Driven by Artificial Intelligence) [21], and the GAMER (Generative Artificial Intelligence Tools in Medical Research) guideline [24]. Leveraging this foundation is scientifically sound and resource efficient. Given the rapid pace of technological development in AI, a multiyear, de novo consensus process would risk producing an obsolete guideline upon publication. Therefore, we synthesized and adapted relevant elements from already existing, established frameworks, creating a focused, cross-disciplinary, and applicable checklist for AI-assisted SLRs.

Second, we extracted all individual checklist items from the selected guidelines for a qualitative content analysis. The guiding question for this analysis was as follows: is this reporting principle relevant and applicable to the use of AI as a methodological tool in an SLR? Each item was evaluated for its relevance to reproducibility, feasibility for authors, and adaptability to the SLR context. Items not considered relevant (eg, those concerning the patient safety of an AI intervention) were excluded. The analysis focused on principles such as the detailed disclosure of AI models, data origins, and the explicit description of human-AI interaction and oversight.

Third, the resulting relevant items were thematically synthesized to identify recurring core concepts (eg, “AI tool identification” and “human-AI interaction”). These thematic clusters were then mapped to the existing structure of the PRISMA 2020 statement to ensure seamless integration and user-friendliness.

### Limitations

We recognize that the proposed checklist has limitations as it results from a systematic synthesis and adaptation, not a formal, broad-based Delphi study or consensus meeting. While its foundation is built on prior consensus, the checklist has not yet undergone this process. Its feasibility was assessed based on expert knowledge, but it has not been validated through a formal user study. Therefore, this work is explicitly intended as a well-founded proposal that provides an immediate solution and can serve as the foundation for a subsequent, community-driven formal consensus process.

### Ethical Considerations

This paper presents a methodological proposal for a reporting guideline and does not involve research on human participants or animals. Therefore, approval from an institutional review board or an ethics committee was not required. The development of this work adheres to the ethical principles for medical research outlined in the World Medical Association Declaration of Helsinki and follows the guidelines on publication ethics of the Committee on Publication Ethics. The use of generative AI during the preparation of this manuscript is disclosed in the Acknowledgments section. The authors retain full responsibility for the scientific integrity and content of this work.

## Results

The previously described method resulted in the PRISMA-trAIce checklist and a corresponding adapted PRISMA flow diagram, which we present in the following sections.

### The PRISMA-trAIce Checklist

The PRISMA-trAIce checklist is an extension to the PRISMA 2020 statement designed to guide the transparent reporting of AI use in SLRs. It comprises 14 items that are categorized according to the structure of an SLR (title, abstract, introduction, methods, results, and discussion) and addresses critical aspects of AI use, from tool identification and human-AI interaction to performance evaluation and limitations.

The complete checklist is presented in Table 1. A detailed explanation of each checklist item, including its rationale, elaboration, sources, and application examples, is provided in Multimedia Appendix 1.

**Table 1.** The PRISMA^a^-trAIce (Preferred Reporting Items for Systematic Reviews and Meta-Analyses–Transparent Reporting of Artificial Intelligence in Comprehensive Evidence Synthesis) checklist for documenting artificial intelligence (AI) tool use in systematic literature reviews (SLRs).

Structural element and item ID		Details
Title		
	P-trAIce T1 - Title	If AI tools played a substantial role in the review process (eg, primary screening, data extraction), consider indicating the use of AI assistance in the title or subtitle.
Abstract		
	P-trAIce A1 - Abstract	Briefly summarize the AI tool(s) used, the SLR stage(s) at which they were applied, and their primary role.
Introduction		
	P-trAIce I1 - Introduction	Briefly state the rationale for choosing to use AI tools for specific tasks in the review (eg, managing large volume of literature, enhancing efficiency, exploring novel methods).
Methods		
	P-trAIce M1 - Protocol and Registration	If specific AI tools or AI-assisted methods were pre-specified in the review protocol, state this and provide details of where the protocol can be accessed. Report any deviations from the protocol regarding AI use.
	P-trAIce M2 - Identification and Access	For each AI tool or system used: a. Specify the name, version number (if applicable), and developer/provider. b. Provide details on how the tool can be accessed (eg, URL, software repository, commercial availability, local instance). c. If a custom-developed AI tool or script was used, describe its core functionality and how it can be accessed or replicated (eg, link to code repository, dataset or base model of a given fine-tuning process).
	P-trAIce M3 - Purpose and Stage of Application	For each AI tool, clearly describe: a. The specific SLR stage(s) where it was applied (e.g., search, screening, data extraction, Risk of Bias assessment, synthesis, drafting). b. The precise task(s) the AI was intended to perform at each stage.
	P-trAIce M4 - Input Data	Describe the input data provided to each AI tool for its operation: a. For tools that learn or are fine-tuned: Describe the origin, nature, and preparation of any data used for training, fine-tuning, or calibration specific to this review. b. For tools applied to review data: Describe the data fed into the tool (eg, search results, abstracts, full-text articles, specific datasets for analysis).
	P-trAIce M5 - Output Data	Describe the output data generated by each AI tool. This description must include the format of the output (eg, structured JSON, plain text, classification labels with confidence scores) and detail any automated post-processing steps applied to the raw output before it was presented for human review or use.
	P-trAIce M6 - Prompt Engineering (if any)	For each LLM^b^/GenAI^c^ tool used, report: a. The full prompt(s) employed for each specific task. If prompts are extensive, provide a detailed description of their structure, key instructions, context provided (eg, inclusion/exclusion criteria, PICO^d^ elements), and any few-shot examples used. Indicate where full prompts can be accessed (eg, supplementary material, repository). b. Key parameters influencing output (eg, model temperature, max tokens, top-p). c. Describe any iterative refinement process for the prompts based on initial outputs or pilot testing.
	P-trAIce M7 - Operational Details and Settings	For AI tools other than LLMs/GenAI (or in addition to prompts for them), describe: a. Key algorithms or models employed by the tool (if known and relevant). b. Specific settings, parameters, or configurations used that could influence performance (eg, thresholds for classification in screening tools, active learning model parameters).
	P-trAIce M8 - Human-AI Interaction and Oversight	Describe the process of human interaction with and oversight of the AI tool(s) at each stage: a. How many reviewers interacted with/validated the AI outputs for each task? b. Did reviewers work independently when validating AI outputs? c. What were the qualifications or training of reviewers for AI-assisted tasks? d. How were AI outputs presented to reviewers? e. What proportion of AI outputs were manually reviewed/verified? f. How were discrepancies between AI and human reviewers, or among multiple human reviewers, resolved? g. Describe any processes for calibrating human reviewers or the AI tool. f. Describe the standard procedure for human verification of AI-generated outputs
	P-trAIce M9 - AI Performance Evaluation (Methods)	Describe methods used to evaluate the AI tool(s) performance for the specific tasks within the review (if applicable and feasible). This may include: a. The reference standard used for evaluation (eg, consensus human decisions). b. The metrics used (eg, accuracy, sensitivity, specificity, precision, recall, F1-score). c. Analyzes conducted to access model bias or rate of erroneous ouputs d. Any pilot testing or validation phases conducted prior to full implementation.
	P-trAIce M10 - Data Governance and Ethics	Describe how data handled by AI tools (input, output, intermediate data) was managed and stored, and any measures taken to ensure data privacy, security, and compliance with copyright or terms of service, especially when using third-party cloud-based AI tools.
Results		
	P-trAIce R1 - Study selection (AI-assisted)	The PRISMA flow diagram and text clearly distinguish between records/reports excluded or included based on AI tool decisions versus human reviewer decisions at each screening stage where AI was used. Report the number of records processed by AI and the outcomes of that processing.
	P-trAIce R2 - AI Performance Metrics (Results)	Report the results of any performance evaluations of the AI tool(s) for the specific tasks within the review (as described in P-trAIce M9). Include quantitative results (see M9) and measures of agreement between AI and human reviewers if assessed.
Discussion		
	P-trAIce D1 - Limitations of AI Use	Discuss any limitations encountered in using the AI tool(s) (eg, technical issues, biases identified, challenges in prompt engineering, unexpected outputs, limitations in AI performance for specific sub-tasks). Discuss how these limitations might have influenced the review process or findings.
	P-trAIce D2 - Implications of AI Use	Briefly discuss the experience of using AI tools in the review, including any perceived benefits (eg, efficiency gains, ability to handle larger datasets) or challenges. Reflect on the usability of the AI tools and the implications for future similar reviews.

a PRISMA: Preferred Reporting Items for Systematic Reviews and Meta-Analyses.

b LLM: large language model.

c GenAI: generative AI.

d PICO: population, intervention, comparison, and outcome.

### The PRISMA-trAIce Flow Diagram

**Figure 1.**
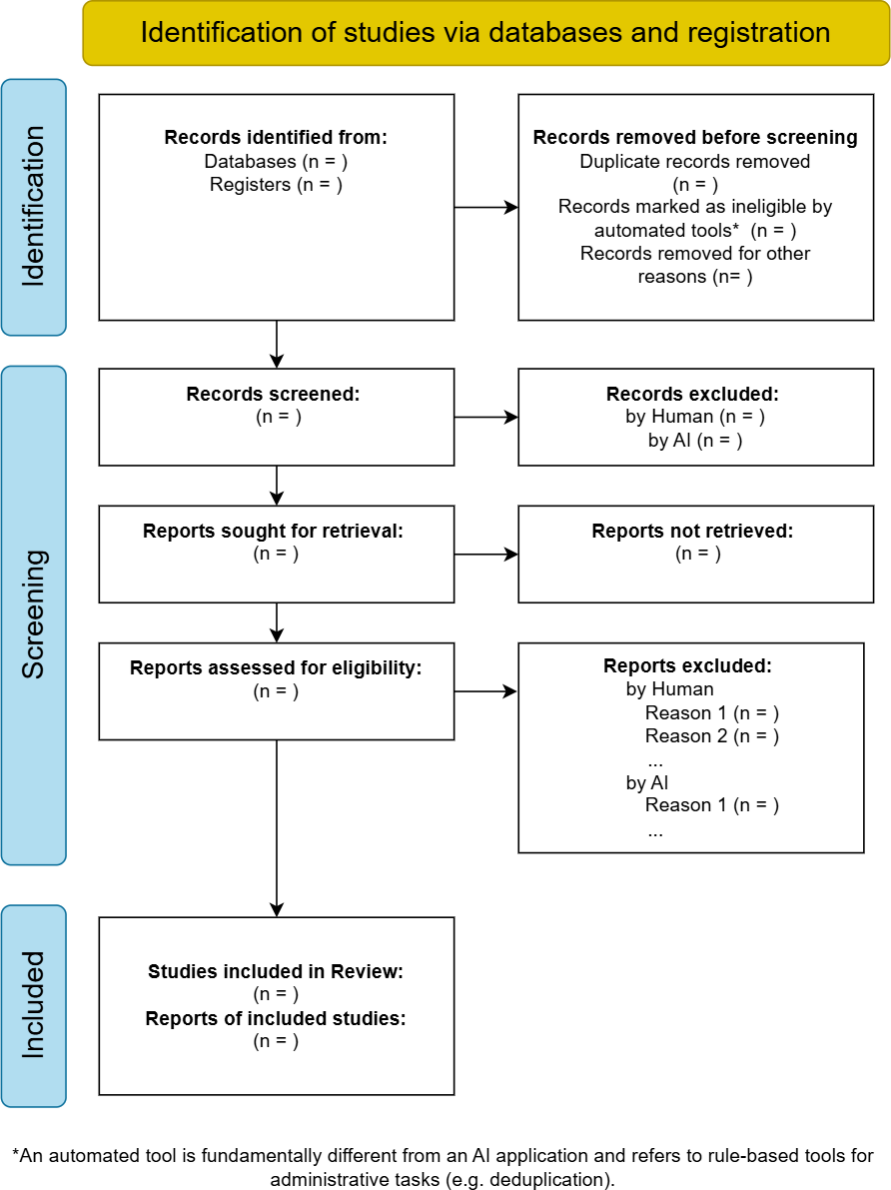
The PRISMA-trAIce (Preferred Reporting Items for Systematic Reviews and Meta-Analyses–Transparent Reporting of Artificial Intelligence in Comprehensive Evidence Synthesis) flow diagram, an adaptation of the standard PRISMA (Preferred Reporting Items for Systematic Reviews and Meta-Analyses) 2020 flowchart. The core modification is the addition of separate fields to distinguish between exclusions made by human reviewers and those made by artificial intelligence (AI) systems, enhancing the transparency of the screening process while preserving the familiar workflow.

To enhance the transparency of the study selection process, the standard PRISMA 2020 flow diagram was adapted (Figure 1). While the original diagram already includes “automation tools,” our proposed version sharpens this concept by distinguishing between rule-based administrative tools (eg, for deduplication) and evaluative AI systems. Therefore, the adapted diagram provides specific fields to report the number of records screened separately by AI systems versus human reviewers and the reasons for exclusion. This visual guide offers a more granular and immediate overview of the AI’s role in the screening process.

## Discussion

### Principal Results

This work’s primary contribution is the PRISMA-trAIce proposal, a checklist designed as an extension to the PRISMA 2020 statement. Its purpose is to provide a standardized framework for the transparent reporting of AI as a methodological tool in SLRs while maintaining ease of use for everyone who is already familiar with the original PRISMA 2020 statement. By systematically synthesizing core principles from established, consensus-based AI reporting guidelines, we offer a robust starting point to address a critical gap in the current landscape of scientific reporting.

### Rationale for a “Living Guideline”

The central challenge in creating reporting guidelines for a field as dynamic as AI is the tension between methodological rigor and timely relevance. A formal consensus process, while it is the standard, risks delivering a meticulously crafted guideline for an already outdated technological landscape. We argue that the immediate risk posed by the nontransparent use of AI tools outweighs the risk of introducing a well-reasoned albeit preliminary guideline. Therefore, our approach is pragmatic, positioning PRISMA-trAIce as a solution that the research community can immediately adopt and refine.

### Limitations

The primary limitation of this proposal is inherent in its nature: it is the result of a systematic adaptation, not a formal, large-scale consensus-building exercise, such as a Delphi study. While grounded in established principles, its items have not yet been empirically validated across diverse research contexts. We do not see this as a shortcoming to be rectified later through traditional means but rather as justification for a more modern, agile approach to standard setting.

Therefore, we propose a community-driven, 2-tiered framework to guide the evolution of PRISMA-trAIce into a living standard. This includes (1) a central, stable anchor on GitHub [25] to serve as the single source of truth for the checklist, enabling transparent, version-controlled development; and (2) a dynamic community hub on Discord to foster rapid and low-friction collaboration. To provide a predictable development cycle, we envision a schedule of annual reviews to incorporate community feedback into future versions. This strategy applies the principles of open-source development to scientific standard setting, ensuring that PRISMA-trAIce can evolve in step with the technology it seeks to describe. We invite experts in evidence synthesis, methodology, and AI to contribute to this initiative. Our long-term vision is to foster a self-sustaining ecosystem. To this end, we are committed to facilitating the establishment of a formal steering committee constituted from dedicated members of the expert community, to which stewardship of the standard will ultimately be transferred.

### Comparison With Prior Work

PRISMA-trAIce occupies a unique and critical niche within the landscape of AI reporting guidelines by providing the first framework specifically designed for AI as a methodological tool in evidence synthesis, a gap not addressed by existing standards. We position our contribution by highlighting 2 key distinctions.

On the one hand, it focuses on AI as a tool, not the subject. Guidelines such as CONSORT-AI [18] and the TRIPOD (Transparent Reporting of a Multivariable Prediction Model of Individual Prognosis or Diagnosis) statements (including TRIPOD-LLM) [20,23] are indispensable for studies in which AI is the subject of the research, for instance, as a diagnostic intervention or a clinical prediction model. These frameworks guide the reporting of the AI’s performance and impact as a study outcome. In contrast, PRISMA-trAIce is designed for the scenario in which AI is part of the research process itself. It guides the reporting on how this tool was used to produce the final systematic review.

On the other hand, it is a discipline-agnostic and SLR-specific framework. While some guidelines offer advice on reporting the use of AI tools, their scope is often domain specific. The GAMER [24] guideline, for example, provides valuable recommendations but is explicitly tailored to the field of medicine. PRISMA-trAIce, by design, is an extension of the universally adopted PRISMA 2020 statement. This makes it inherently discipline agnostic and directly integrable into the established workflow of systematic reviews across any field, from engineering to social sciences. Its structure is mapped directly onto the sections of an SLR, a level of specificity for the evidence synthesis process that other AI reporting guidelines do not provide.

Therefore, by focusing on AI as a methodological tool in a discipline-agnostic and SLR-specific manner, PRISMA-trAIce complements existing guidelines and addresses a critical, unmet need for transparency in the next generation of evidence syntheses.

### Conclusions

The integration of AI into systematic reviews marks a disruptive moment in evidence synthesis, promising efficiency but demanding a renewed commitment to rigor. The current lack of a standardized reporting framework represents a critical threat to the transparency and trustworthiness of these AI-assisted reviews. The proposed PRISMA-trAIce checklist provides a foundational tool to address this challenge, aiming to immediately improve transparency while paving the way for a formal, community-endorsed standard.

## Supplementary material

10.2196/80247 Multimedia Appendix 1 The PRISMA-trAIce (Preferred Reporting Items for Systematic Reviews and Meta-Analyses–Transparent Reporting of Artificial Intelligence in Comprehensive Evidence Synthesis) statement—elaboration, explanation, and examples.

## Abbreviations

AI: artificial intelligence
CONSORT-AI: Consolidated Standards of Reporting Trials–Artificial Intelligence
DECIDE-AI: Developmental and Exploratory Clinical Investigations of Decision Support Systems Driven by Artificial Intelligence
EQUATOR: Enhancing the Quality and Transparency of Health Research
GAMER: Generative Artificial Intelligence Tools in Medical Research
PRISMA: Preferred Reporting Items for Systematic Reviews and Meta-Analyses
PRISMA-AI: Preferred Reporting Items for Systematic Reviews and Meta-Analyses–Artificial Intelligence Extension
PRISMA-trAIce: Preferred Reporting Items for Systematic Reviews and Meta-Analyses–Transparent Reporting of Artificial Intelligence in Comprehensive Evidence Synthesis
SLR: systematic literature review
SPIRIT-AI: Standard Protocol Items: Recommendations for Interventional Trials–Artificial Intelligence
TRIPOD: Transparent Reporting of a Multivariable Prediction Model of Individual Prognosis or Diagnosis
TRIPOD-AI: Transparent Reporting of a Multivariable Prediction Model of Individual Prognosis or Diagnosis–Artificial Intelligence
TRIPOD-LLM: Transparent Reporting of a Multivariable Prediction Model of Individual Prognosis or Diagnosis–Large Language Model
