# Supplementary material for: Transparent Reporting of AI in Systematic Literature Reviews: Development of the PRISMA-trAIce Checklist
Source: JMIR AI. 2025 Dec 10;4:e80247. doi: 10.2196/80247 (PMC12694947; doi:10.2196/80247)
Supplement: Multimedia Appendix 1 [file ai-v4-e80247-s001.docx]

## Multimedia Appendix 1: The PRISMA-TRAICE Statement – elaboration, explanation, sources, and examples

The ongoing integration of AI into the methodology of systematic reviews underscores the need for clear and specific reporting standards to ensure the continued integrity and utility of research. To address this, we have developed PRISMA-trAIce, a dedicated reporting guideline structured as an extension of the PRISMA 2020 statement.

The fundamental aim of PRISMA-trAIce is to provide authors with a clear framework to enhance the transparency, methodical rigor, reproducibility, and overall trustworthiness of systematic reviews that employ AI tools. To facilitate this, the following sections explain on each item within the PRISMA-trAIce checklist, explaining its rationale, intended scope and given source from other AI Guidelines. To demonstrate the checklist's practical application, all examples are cohesively drawn from a single, fictitious systematic review titled 'Effectiveness of Mindfulness Interventions for Workplace Stress'. This narrative approach is designed to illustrate how the checklist items integrate into the workflow of a real-world systematic review.

To guide authors in applying the checklist, each PRISMA-trAIce item is assigned one of three recommendation levels. The rationale for these levels is designed to balance the need for comprehensive transparency with practical applicability. Items designated as **Mandatory** are essential for a baseline assessment of the review's validity and reproducibility. **Recommended** or **Highly Recommended** applies to items that significantly enhance the transparency and depth of the report. Finally, **Optional** items relate to sections of the paper where forcing AI-specific information could conflict with the primary purpose of that section, for instance, the conciseness of a title.

#### Title

**Item T1 Title (Optional):** If AI tools played a substantial role in the review process, such as for primary screening or data extraction, indicating this assistance in the title or subtitle is recommended. This practice enhances immediate transparency for readers regarding key methodological aspects and allows for the easier identification of AI-assisted reviews.

Example: "Effectiveness of Mindfulness Interventions for Workplace Stress: A Systematic Review Using AI-Assisted Literature Screening.

Sources: CONSORT-AI 1a; DECIDE 1; SPIRIT-AI 1 (i); TRIPOD-AI 1; TRIPOD-LLM 1

#### Abstract

**Item A1: Abstract (Optional):** The abstract should briefly summarize the AI tool(s) used, the SLR stage(s) at which they were applied, and their primary role. This provides a concise overview of the AI involvement, similar to how other key methodological features are highlighted in an abstract.

Example: " Methods: Following a search across five databases yielding 15,230 records, we employed ASReview (v1.0) with a Naive Bayes active learning model for semi-automated screening of titles and abstracts. All potentially relevant records were subsequently verified by two independent human reviewers."

Sources: CONSORT-AI 1b; TRIPOD-LLM 2; DECIDE I

#### Introduction

**Item I1: Introduction (Recommended):** If applicable, the rationale for using AI tools for specific tasks in the review should be briefly stated (e.g., managing a large volume of literature, enhancing efficiency, or exploring novel methods). Providing this information contextualizes the methodological choices involving AI.

Example: "The body of literature on mindfulness for workplace stress is expansive and growing rapidly. To manage the large volume of search results efficiently while maintaining methodological rigor, we integrated an AI-assisted screening tool into our review process."

Sources: CONSORT-AI 2a; DECIDE 2; SPIRIT-AI 6a; TRIPOD-AI 3a, b; TRIPOD-LLM 3a, b & 4

#### Methods

**Item M1: Protocol and Registration (Mandatory):** If the use of specific AI tools or AI-assisted methods was pre-specified in the review protocol, this should be stated, along with details on where the protocol can be accessed. Any deviations from the protocol regarding AI use must also be reported. Mentioning the AI tools used in the protocol reduces the risk of p-hacking and post-hoc justifications. It also prevents several AI tools from being used in parallel and only those tools that had a positive impact being documented.

Example: " Our review protocol was pre-registered on PROSPERO (CRD42025123456) and detailed the planned use of ASReview for title and abstract screening. We deviated from the protocol by additionally employing GPT-5 to assist in the preliminary data extraction from included studies, as a pilot test showed this to be more efficient than a fully manual process."

Sources: CONSORT-AI 23 & 24; DECIDE-AI III; SPIRIT-AI 2a; TRIPOD-AI 18c, d; TRIPOD-LLM 14c, d

**Item M2: AI Tool(s) - Identification and Access (Mandatory):** For each AI tool or system used, authors should specify its name, version number, and developer/provider. Furthermore, details must be provided on how the tool can be accessed. If a custom-developed tool was used, its core functionality and the means by which it can be accessed or replicated must also be described.

Example: " For title and abstract screening, we used the open-source software ASReview (v1.0, ASReview contributors), accessible at https://asreview.nl/. For data extraction assistance, we used GPT-5 (OpenAI, August 2024 version), accessed via its web-based user interface."

Sources: CONSORT-AI 5 (i); DECIDE 4a & 11; GAMER 2 & 4; SPIRIT-AI 11a (i) TRIPOD-AI 22; TRIPOD-LLM 6a, 6b, 14f

**Item M3: AI Tool(s) - Purpose and Stage of Application (Mandatory):** For each AI tool, authors should clearly describe both the specific SLR stage(s) where it was applied (e.g., search, screening, data extraction) and the precise task(s) the AI was intended to perform at each stage.

Example: " ASReview (v1.0) was used during the title and abstract screening stage to prioritize records for human review based on relevance. GPT-5 was subsequently applied during the data extraction stage to populate a predefined data table with information such as study design, participant count, and intervention type from the full-text articles."

Sources: CONSORT-AI 4b; DECIDE 5a; GAMER 5 & 6; SPIRIT-AI 6a (i); TRIPOD-AI 3b

**Item M4: AI Tool(s) - Input Data (Mandatory):** Authors must describe the input data provided to each AI tool for its operation. For tools that were trained or fine-tuned, this includes describing the training data. For tools applied to review data, the data fed into the tool (e.g., search results, abstracts) must be described.

Example: " To calibrate the ASReview active learning model, an initial set of 100 abstracts, randomly selected and screened by two reviewers (50 included, 50 excluded), served as the training data. The remaining 15,130 unscreened records (.ris format) were then imported. For GPT-5, the input consisted of the full-text PDF of each included study."

Sources: CONSORT-AI 5a, 7; DECIDE 4b; SPIRIT-AI 10 (ii) & 11a (ii & iii); TRIPOD-AI 5; TRIPOD-LLM 5

**Item M5: AI Tool(s) - Output Data (Mandatory):** Authors should describe the output data generated by each AI tool, including its format (e.g., structured JSON, classification labels with confidence scores) and any automated post-processing steps applied.

Example: " ASReview generated a ranked list of all records, assigning each a relevance score between 0.0 and 1.0. The output of the GPT-5 data extraction prompt was a structured JSON object for each study. No automated post-processing was applied before human verification."

Sources: CONSORT-AI 5 (v); DECIDE 4(c); SPIRIT-AI 11a (v); TRIPOD-AI 15; TRIPOD-LLM 6d

**Item M6: AI Tool(s) - Prompt Engineering (if LLMs/GAI used) (Mandatory):** For each LLM/GAI tool used, the prompt engineering process must be reported, including the full prompt(s) or a detailed description, key parameters (e.g., temperature), and any iterative refinement process.

Example: " For data extraction using GPT-5, a detailed prompt was engineered. The base prompt was: 'You are a research assistant. From the following article on mindfulness, extract these data points and return them as a JSON object: [list of fields like 'study_design', 'participant_count', etc.].' The model temperature was set to 0.1 for consistency. The full prompt is available in Supplementary Material S1."

Sources: GAMER 3; TRIPOD-LLM 9, 11

**Item M7: AI Tool(s) - Operational Details and Settings (Highly Recommended):** For AI tools other than LLMs, key operational settings should be described, such as the algorithms used or specific parameters that could influence performance.

Example: " ASReview was configured to use the Naive Bayes (NB) classifier with default TF-IDF feature extraction. The balance strategy was 'dynamic,' and the query strategy was 'max uncertainty.'"

Sources: TRIPOD-AI 12b & f; TRIPOD-LLM 6e; TRIPOD-LLM 11

**Item M8: Human-AI Interaction and Oversight (Mandatory):** The process of human interaction with and oversight of the AI tool(s) must be described comprehensively for each stage of its application. This includes reporting on the number of reviewers who validated the AI outputs and whether they worked independently. Their qualifications and any specific training received for these AI-assisted tasks should be stated. Furthermore, the description must clarify how the AI-generated outputs were presented to the reviewers and what proportion of these outputs was manually verified. The standard procedure for resolving discrepancies, whether between the AI and a human reviewer or among multiple human reviewers, needs to be detailed, as should any processes used for the calibration of reviewers or the AI tool itself.

Example: " Two researchers independently screened all abstracts that ASReview ranked in the top 20% of relevance (n=3,046). To validate the AI's exclusions, a random 5% sample of the remaining records (n=604) was also dual-screened. Disagreements were resolved by a senior reviewer. For data extraction, the JSON output from GPT-5 for every study was independently verified by one researcher against the original PDF."

Sources: CONSORT-AI 5 (iv); DECIDE 6, 7 & 10a, 12; GAMER 7; SPIRIT-AI 11a (iv); TRIPOD-LLM 7b

**Item M9: AI Performance Evaluation (Mandatory):** If applicable, the methods used to evaluate the performance of the AI tool(s) should be described, including the reference standard used (e.g., consensus human decisions), the metrics employed (e.g., accuracy, sensitivity, precision), any specific analyses conducted to assess for biases or the rate of erroneous outputs ("hallucinations"), and any pilot testing or validation phase prior to full implementation.

Example: " To evaluate the performance of the ASReview-assisted screening, we calculated the number of relevant studies missed in the random 5% sample of low-ranked records. We also calculated inter-rater reliability (Cohen’s Kappa) for the human verification of the GPT-5 data extraction."

Sources: DECIDE 4a, 12; TRIPOD-AI 9; TRIPOD-LLM 7a, c & e

**Item M10: Data Management and Ethics (Recommended):** Authors should describe how data handled by AI tools were managed, stored, and measures taken to ensure data privacy, security, and compliance with copyright or terms of service.

Example: " The initial search results were stored on our institution's secure server. For the use of the cloud-based GPT-5 API, we ensured compliance with our institution's data security policies for handling published research articles.“

Sources: DECIDE 8; GAMER 8; SPIRIT-AI 19, 27 & 29

#### Results

**Item R1: Study selection (Mandatory):** In the PRISMA flow diagram and text, authors must clearly distinguish between records included or excluded by AI tool decisions versus those decided by human reviewers. The number of records processed by the AI must be reported. If an AI system is advanced enough to base its decision on a reason, this reason should be listed too.

Example: " The PRISMA flow diagram notes that all 15,230 records were prioritized via ASReview. The 'Records excluded' box (n=14,984) is annotated to clarify that this human screening was based on the AI-prioritized list, with the top 20% being fully reviewed."

Source: PRISMA 2020 Adaptation (New)

**Item R2: AI Performance Metrics (Mandatory):** The results of any performance evaluations of the AI tool(s) should be reported, including measures of agreement between the AI and human reviewers if assessed.

Example: " The validation of the ASReview process revealed that one relevant study was missed in the 5% random sample of low-ranked records, resulting in an estimated workload reduction of ~80% with a recall of over 99%. The inter-rater reliability for the verification of GPT-5 data extraction was high (Cohen’s Kappa = 0.92)."

Sources: CONSORT 19; TRIPOD-AI 20c, 23a, 24; TRIPOD-LLM 17

#### Discussion

**Item D1: Limitations of AI Use (Recommended):** The discussion should address any limitations encountered in the use of the AI tool(s), such as technical issues, identified biases, or challenges in prompt engineering, or performance limitations such as observed biases or erroneous outputs ("hallucinations"). Authors should discuss how these limitations might have influenced the review’s findings and describe any steps taken to mitigate their impact.

Example: " A limitation of ASReview was its initial difficulty in distinguishing between workplace and clinical settings for mindfulness interventions, which required an expanded set of 100 training examples to calibrate effectively. Furthermore, the GPT-5 model occasionally failed to extract the specific 'outcome measures' correctly, necessitating careful manual verification and correction."

Sources: CONSORT-AI 19; DECIDE 14a; CONSORT-AI 20; GAMER 9; TRIPOD-AI 26; TRIPOD-LLM 19b, c

**Item D2: Implications of AI Use (Optional):** The discussion should briefly address the experience of using AI tools, including perceived benefits or challenges, and reflect on the implications for future similar reviews.

Example: " The use of ASReview accelerated the screening process significantly, reducing the estimated time for this phase from over a month to two weeks and saving approximately 80 person-hours of work."

Source: CONSORT-AI 5 (vi); DECIDE-AI 7 & 14; GAMER 9; TRIPOD-AI 27; TRIPOD-LLM 19a, f & g
